# Opportunities and barriers for providing HIV testing through community health centers in mainland China: a nationwide cross-sectional survey

**DOI:** 10.1186/s12879-019-4673-0

**Published:** 2019-12-16

**Authors:** Jason J. Ong, Ming Hui Peng, William W. Wong, Ying-Ru Lo, Michael R. Kidd, Martin Roland, Shan Zhu Zhu, Sun Fang Jiang

**Affiliations:** 1 Central Clinical School, Monash University, Melbourne, Australia; 2 London School of Hygiene and Tropical Medicine, London, UK; 3 General Practice Department, Zhongshan Hospital Fudan University, 180, Fenlin Road, Xuhui District, Shanghai, 200032 China; 4 Department of Family Medicine and Primary Care, University of Hong Kong, Hong Kong, China; 5 HIV, Hepatitis and Sexually Transmitted Infections Unit Division of Communicable Diseases, World Health Organization Regional Office for the Western Pacific, 1000 Manila, Philippines; 6 Department of Family and Community Medicine, University of Toronto, Toronto, Canada; 7 Southgate Institute for Health, Equity and Society, Flinders University, Adelaide, Australia; 8 Institute of Public Health, University of Cambridge School of Clinical Medicine, Cambridge, UK

**Keywords:** HIV, Primary care, Community health centre, China, Testing

## Abstract

**Background:**

Primary care may be an avenue to increase coverage of HIV testing but it is unclear what challenges primary healthcare professionals in low- and middle-income countries face. We describe the HIV testing practices in community health centres (CHCs) and explore the staff’s attitude towards further development of HIV testing services at the primary care level in China.

**Methods:**

We conducted a national, cross-sectional survey using a stratified random sample of CHCs in 20 cities in 2015. Questionnaires were completed by primary care doctors and nurses in CHCs, and included questions regarding their demographics, clinical experience and their views on the facilitators and barriers to offering HIV testing in their CHC. Multivariate logistic regression was conducted to examine the association between staff who would offer HIV testing and their sociodemographic characteristics.

**Results:**

A total of 3580 staff from 158 CHCs participated. Despite the majority (81%) agreeing that HIV testing was an important part of healthcare, only 25% would provide HIV testing when requested by a patient. The majority (71%) were concerned about reimbursement, and half (47%) cited lack of training as a major barrier. Almost half (44%) believed that treating people belonging to high-risk populations would scare other patients away, and 6% openly expressed their dislike of people belonging to high-risk populations. Staff who would offer HIV testing were younger (adjusted odds ratio (aOR) 0.97 per year increase in age, 95% confidence interval (CI):0.97–0.98); trained as a doctor compared to a nurse (aOR 1.79, 95%CI:1.46–2.15); held a bachelor degree or above (aOR 1.34, 95%CI:1.11–1.62); and had previous HIV training (aOR 1.55, 95%CI:1.27–1.89).

**Conclusions:**

Improving HIV training of CHC staff, including addressing stigmatizing attitudes, and improving financial reimbursement may help increase HIV testing coverage in China.

## Background

In China, HIV affects less than 1% of the general population, but is concentrated in key populations such as men-who-have-sex-with-men (MSM) where prevalence has been reported to be as high as 8% in 2015, and with the total number of people diagnosed with HIV infection reaching 880,000 [1, 2]. The Joint United Nations Programme on HIV/AIDS (UNAIDS) estimates that approximately 54% of people living with HIV (PLHIV) worldwide still do not know their HIV status [3]. Hence, those who are not diagnosed and not receiving antiretroviral therapy (ART) could act as a reservoir for forward transmission of HIV [4]. Increasing the frequency and testing of HIV, especially for members of key populations, is needed if we are to meet the UNAIDS goal of 90–90-90 by 2020 i.e. 90% of HIV infections should be diagnosed; 90% of those people diagnosed should be treated with antiretroviral therapy (ART); and, 90% of those receiving ART would have their viral load suppressed [5].

Despite China having 23,043 laboratories and clinics for HIV Voluntary Counselling and Testing (VCT) [6], nearly all are affiliated with the China Center for Disease Control and Prevention (CDC) or with major urban hospitals. High-risk populations, such as MSM, people who inject drugs, and sex workers and their clients, are generally reluctant to attend these official facilities, resulting in under-diagnosis or delays in HIV testing [7–9]. Other than accessibility, the attitude towards HIV testing both from health professionals and members of high-risk populations can significantly affect testing uptake rates. In China, as in many other parts of the world, fear of stigma and discrimination and concerns about confidentiality are significant barriers to HIV testing [10–12]. In particular, the attitude of some Chinese health professionals about HIV-associated shame and discrimination, as well as fears of being infected, have been strongly correlated with the lack of offering VCT, prevention and treatment [13–18].

There is an opportunity to increase HIV testing in China by encouraging testing through Community Health Centres (CHCs). “The guidance about the development of urban community health services under the State Council”, published in Mainland China in 2006 [19], has led to the establishment of an extensive network of CHCs in cities and township health centers in rural areas throughout China [20]. In 2011, China enacted a social insurance law to strive for equal access to basic primary health services and access to public hospitals for both urban and rural residents [21]. A nationwide survey of CHCs found that the median number of residents and internal migrants served was 61,000 with CHCs reporting a median of 41,100 individuals attending a CHC within a one-year period [22]. By 2014, more than 8000 CHCs were providing services to over 500 million patients [20], demonstrating that CHCs have become an important integral part of the Chinese healthcare system. In two neighbouring countries, Cambodia and Laos, the provision of HIV testing and information sessions in primary care settings has contributed to the success of improved early detection and treatment of PLHIV [23].

The purpose of this study was to describe the current HIV testing practices by CHC staff in Mainland China and to explore their attitude towards further development of such services at the primary care level. We examined what factors were associated with the likelihood of offering HIV testing to patients who requested it. This information is important for policymakers and healthcare providers, and also provides a foundation for planning further interventional studies in primary healthcare in China.

## Methods

### Study design

Regarding sample size calculation, our literature review did not find any studies reporting the proportion of primary health professionals in China that would test for HIV, so we used the outcome of a health professional being willing to manage patients living with HIV as 70% [24]. With 2% margin error and 95% confidence level, a minimum of 2160 health professionals was needed. Assuming a response rate of 60% and an assumed 20 surveys from each CHC, 3600 surveys were sent out between September and December 2015.

Three-stage stratified random sampling was used to select a national sample of CHCs in Mainland China. The sampling frame was divided into Eastern, Central, and Western regions according to the China Health Statistics Yearbook 2015 [20]. We numbered the provinces then used the tool “sample” in Excel to randomly select two provinces from each region and two municipalities from four municipalities (Beijing, Shanghai, Chongqing and Tianjin). A total of eight provinces/municipalities were selected (East Region: Liaoning, Zhejiang; Central Region: Shanxi, Anhui; West Region: Yunnan, Sichuan; Municipalities: Shanghai, Chongqing). Then the capital city and an additional two district-level cities (i.e. second-level administrative subdivision) were randomly selected resulting in 20 cities altogether. Three country-level cities (i.e. third-level administrative subdivision) were selected from each of the above-mentioned cities or municipalities. Nine CHCs with an urban-to-suburban ratio of 2:1 to represent the current concentration of CHCs were randomly selected to participate within a city, resulting in a sample of 180 CHCs in total. The chairpersons of each regional General Practice Associations were contacted to organise the administration of the survey while the research team provided training, guidance and quality management of the surveys.

### Study population

The primary care team in China typically consist of doctors (including traditional Chinese practitioners), nurses and pharmacists. A few also include physiotherapists, clinical psychologists or social workers. The hierarchy of the titles are achieved by CHC staff after passing the qualifying examination and fulfilling a number of stringent criteria including continuous medical education (CME) requirements, written examinations and publication of research manuscripts. More details on the structure and organization of care in China’s CHCs has been reported elsewhere [22]. All medical doctors (except those that exclusively practice Traditional Chinese Medicine) and nurses working in selected CHCs were asked to fill out a questionnaire after a written informed consent was signed. CHC staff were asked about their sociodemographic characteristics; experience of testing for HIV at the CHC; training in HIV testing and management; attitude towards offering HIV testing; and specifically, attitudes towards offering HIV tests to members of key populations. HIV testing can be either provider-initiated or patient-initiated. Provider-initiated HIV testing in China is recommended for members of key populations and pregnant women, and is not to be coercive (i.e. testing is voluntary and providers will obtain informed consent and offer pre-test counselling).

### Measures

Current practice of HIV testing by CHC staff was determined by this question: “What will you do if someone requested an HIV test?” with options of “Tell them you know nothing about HIV and send them away”, “Tell them to come back to see another colleague at your CHC”, “Tell them to go to hospital”, or “Tell them to go to the CDC”. Participants could pick more than one option. Whether CHC staff had ever received training in HIV was assessed in the following domains: pre- and post-test counseling, HIV clinical diagnosis, treatments and nursing for PLHIV, and HIV prevention. We asked about barriers to provide HIV testing in CHC with options of “lack of training”, “lack of financial incentive”, “lack of support from senior colleagues or management”, “not interested” or “other”. We also asked about resources needed to help facilitate HIV testing in CHC with options of “more training”, “detailed guidelines and manuals”, “better support from local hospitals”, “direct hotline to specialists” and “other”. The provider attitude questions for HIV testing were adapted from the US Centres for Disease Control and Prevention ‘Evaluation Toolkit: Patient and Provider Perspectives about Routine HIV Screening in Health Care Settings’ and asked in a Likert-scale format [25]. The full survey is provided in Additional file 1.

### Statistical analysis

Data were double entered, checked, and validated in Epidata 3.1, and analyzed using STATA 13.0 (StataCorp, College Station, TX, USA). Descriptive analysis was used to summarize the demographics of the study population stratified by profession (doctor, nurse) (Table 1), and the proportion who reported ever receiving training for HIV (Table 2). We assessed for geographical heterogeneity in willingness to test for HIV in CHCs using a chi-squared test. These results were stratified according to province/municipality in Additional file 1: Table S1 and Additional file 1: Table S2. The percentage of staff who agreed with statements related to the faciliators, barriers and attitude towards HIV testing is presented in Table 3. Multivariable logistic regression was used to explore the characteristics of staff who would offer HIV testing when requested by patients (Table 4). We included independent variables with a univariable analysis *p* value of less than 0.20 in the multivariable model. The importance of each variable was assessed using a *p* value of its Wald statistic. Variables that were statistically insignificant (*p* > 0.05) were sequentially eliminated from the model. The final model was adjusted for province, age, occupation, highest education level, and training in HIV.

**Table 1:** Demographic characteristics of CHC staff in China

	Total n/N	% (95% CI)	Doctor n/N	% (95% CI)	Nurse n/N	% (95% CI)
Median age (IQR)		35 (28–43)		38 (32–46)		31 (26–39)
Female	2818/3478	81 (80–82)	1025/1675	61 (59–64)	1793/1803	99 (99–100)
Highest qualification						
Graduate degree and higher	1469/3555	41 (40–43)	940/1724	55 (52–57)	529/1831	29 (27–31)
Title						
Senior	226/3526	6 (6–7)	180/1711	11 (9–12)	46/1815	3 (2–3)
Intermediate	1176/3526	33 (32–35)	705/1711	41 (39–44)	471/1815	26 (24–28)
Junior	1841/3526	52 (51–54)	683/1711	40 (38–42)	1158/1815	64 (62–66)
None	283/3526	8 (7–9)	143/1711	8 (7–10)	140/1815	8 (7–9)

*CHC* Community Health Centre, *CI* Confidence interval, *IQR* Interquartile range

**Table 2:** HIV-related training amongst CHC doctors and nurses

	Total n/N	% (95% CI)	Doctor n/N	% (95% CI)	Nurse n/N	% (95% CI)
Pre- and post-test counseling	1185/3443	34 (33–36)	640/1670	38 (36–41)	545/1773	31 (29–33)
HIV clinical diagnosis	1286/3435	37 (36–39)	738/1670	44 (42–47)	548/1765	31 (29–33)
Treatments and nursing for PLHIV	1133/3432	33 (31–35)	540/1660	33 (30–35)	593/1772	34 (31–36)
HIV prevention	1945/3483	56 (54–58)	966/1685	57 (55–60)	979/1798	54 (52–57)

*CHC* Community Health Centre, *CI* Confidence interval

**Table 3:** Facilitators, barriers and attitudes to provide HIV testing in CHC (*n* = 3580)

	Total % (95% CI)	Doctors % (95% CI)	Nurses % (95% CI)
Resources needed before HIV testing offered at CHC			
- More medical training	41 (39-43)	39 (36-41)	43 (40-45)
- Guidelines	19 (18-21)	19 (17-21)	19 (17-21)
- Better support from hospitals	12 (11-13)	12 (11-14)	12 (10-13)
- Direct hotline to specialists	3 (3-4)	3 (2-4)	3 (3-4)
- Other	2 (2-2)	2 (1-3)	2 (1-3)
- Already provide testing	23 (21-24)	24 (22-26)	21 (19-23)
Worries about offering HIV testing to high-risk populations			
- Drive other patients away	44 (42-46)	42 (39-44)	46 (43-48)
- Patients too difficult to manage	60 (58-61)	60 (58-63)	59 (56-61)
- Get infected by them	38 (37-40)	32 (29-34)	44 (42-47)
- Not interested	6 (5-7)	6 (5-8)	6 (5-7)
- Hate these people	7 (6-8)	6 (5-7)	7 (6-9)
- Others	6 (5-7)	7 (6-8)	4 (3-5)
Attitude towards offering HIV testing in CHC			
- HIV testing is an important part of healthcare	81 (79-83)	83 (81-85)	84 (82-86)
- Concerned about costs and reimbursement for HIV testing	80 (79-82)	79 (77-81)	81 (79-83)
- Concerned that patients will be offended by being offered HIV testing	83 (82-85)	83 (81-85)	83 (81-85)
- Comfortable discussing HIV testing	56 (55-58)	55 (53-58)	57 (55-60)
- Patients receive adequate pre-test information for HIV testing	82 (80-83)	81 (79-83)	82 (81-84)
- Patients received adequate post-test information for HIV testing	84 (83-85)	83 (81-85)	84 (82-86)
- HIV testing is voluntary	73 (71-75)	74 (72-76)	72 (70-74)
- Patients do not expect to be offered HIV testing	64 (62-66)	65 (62-68)	63 (60-65)

*CHC* Community Health Centre, *CI* Confidence interval

**Table 4:** Logistic regression analyses of the characteristics of CHC staff who would offer HIV testing on request (*n* = 3110)

	Offer HIV testing *n* (%)	Crude OR (95% CI)	*p* value	Adjusted OR* (95% CI)	*p* value
Age (per year)	-	0.97 (0.97–0.98)	< 0.01	0.97 (0.96–0.98)	< 0.01
Doctor	487 (55)	1.46 (1.25–1.70)	< 0.01	1.79 (1.46–2.15)	< 0.01
Nurse (ref)	390 (44)				
Graduate degree or above	472 (54)	1.26 (1.08–1.47)	< 0.01	1.34 (1.11–1.62)	< 0.01
Below graduate degree (ref)	396 (46)				
Years in current work (per year)	-	0.98 (0.97–0.99)	< 0.01	-	-
Received training in HIV	589 (76)	1.75 (1.46–2.11)	< 0.01	1.55 (1.27–1.89)	< 0.01
No training (ref)	187 (24)				

*CI* Confidence interval, *OR* Odds ratio, *Ref* Reference group

*Adjusted for province, age, occupation, highest education level, and training in HIV

## Results

**Fig. 1:**
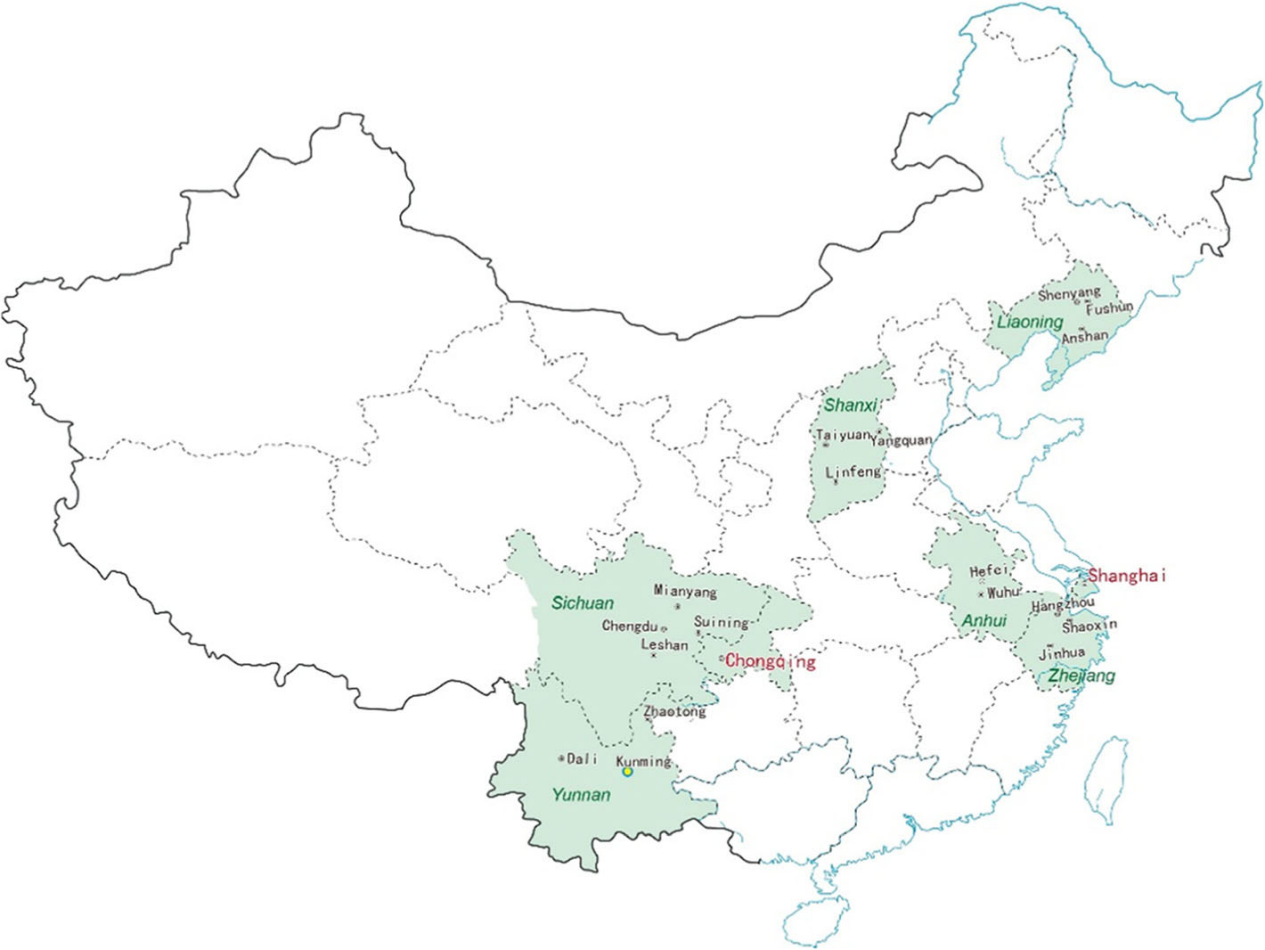
The geographical distribution of cities where data was received from CHCs (provinces in green, cities in black and municipalities in red text)

Out of the 189 CHCs approached, 158 (83.6%) participated in the study. Fig. 1 shows the geographical distribution of the cities with complete CHC data. A total of 4146 questionnaires were distributed with 3698 (89.2%) questionnaires returned. A small number of questionnaires (*n* = 198) were invalid due to excessive missing data. The final sample included 1734 doctors and 1846 nurses from 158 CHCs.

The median number of residents served by each CHC was 50,000 (interquartile range (IQR) 30,000-96,000) with an additional median number of an internal migrant population of 11,100 (IQR 5000–30,000). In the previous year, the median number of patients who visited each CHC was 41,100 (IQR 12,000–163,600). Nearly half (44%; 95% CI: 36–53%) of the CHCs had a protocol for HIV testing, and 14% (95% CI: 7–22%) for HIV treatment.

Table 1 summarizes the demographic characteristics of the CHC medical staff. If HIV testing was requested by a patient, 54% (95% CI: 52–55) would refer the patient to a CDC for the test, 15% (95% CI: 14–17) would tell them to go to a hospital, 4% (95% CI: 3–5) would refer to another colleague at the CHC and 0.8% (95% CI: 0.5–1.1) would tell them to go away. Only 25% (95% CI: 24–27) would provide HIV testing for the patient at the CHC. This varied significantly according to the location of the CHC: western districts (Sichuan (36%), and Yunnan (42%)); central districts (Shanxi (11%), Anhui (22%)); eastern districts (LiaoNing (17%), Zhejiang (33%)) and the two municipalities (Shanghai (12%), Chongqing (25%) (*p* < 0.001). About half (51, 95% CI: 49–52) felt comfortable discussing HIV testing with patients, 52% (95% CI: 51–54%) felt confident that they could ask their patients about their sexual history, and 47% (95% CI: 45–49%) felt confident in asking about their patient’s sexual orientation. Half of CHC staff (51, 95% CI: 49–53) thought it would be difficult to guarantee confidentiality to patients having an HIV test.

A minority of doctors had diagnosed (6, 95% CI: 5–8), or provided clinical care (4, 95% CI: 3–5) to people living with HIV within the preceding month. A further 6% (95% CI: 5–7) had provided healthcare to patients whom they suspected of having HIV. They also reported consulting with people who inject drugs (4, 95% CI: 3–5), female sex workers (5, 95% CI: 4–7), male sex workers (2, 95% CI: 2–3), men who have sex with men (2, 95% CI: 1–3) and transgender individuals (1, 95% CI: 0–1) in the preceding month. Whilst 91% (95% CI: 90–92) of medical staff received regular CME, only a third had received HIV-specific training (Table 2). Table 3 summarises the facilitators, barriers and atttitudes towards offering HIV testing in CHC. Whilst the majority agreed that HIV testing was an important part of healthcare, multiple barriers for offering HIV testing at CHCs were identified. The majority of CHC staff were concerned about not receiving fees for testing and reimbursement (80%), and were concerned that patients will be offended by being offered HIV testing (83%, Table 3).

The main barriers for providing HIV testing specifically to people from key populations were: 60% (95% CI: 58–61) felt it was too difficult to manage HIV, 44% (95% CI: 42–45%) were worried that treating people with HIV patients would scare other patients away and 38% (95% CI: 37–40%) were worried they might get infected with HIV through treating PLHIV. Six percent (95% CI: 6–8) of medical staff simply disliked the members of high-risk population or were not at all interested in dealing with HIV (6, 95% CI: 5–7).

The multivariate analyses in Table 4 shows that CHC staff who provided HIV testing were more likely to be younger (adjusted odds ratio (aOR) 0.97 per year increase in age), trained as a doctor compared to a nurse (aOR 1.79), hold a graduate degree or above (aOR 1.34), or have had received specific HIV training (aOR 1.55).

## Discussion

This is the first national study of CHCs in China to examine current practices of HIV testing in CHCs, the attitude of staff, and the perceived facilitators and barriers when providing HIV testing to patients attending CHCs including to key populations. We found that only a quarter of CHC staff would provide HIV testing even when a patient requested it. Of note, less than half of doctors and a third of nurses have received training on HIV diagnoses, indicating that more widespread education for CHC staff is urgently needed if HIV test coverage in China is to increase. Several barriers were identified which will need to be addressed before encouraging other CHCs to provide HIV testing. These included providing more education and clinical resources, addressing financial concerns and stigmatizing attitudes (e.g. nearly half the respondents believed that people from high-risk populations would scare other patients away, and over a third were worried that they could be infected with HIV). Despite these perceived barriers, the majority of staff agreed that HIV testing was an important part of healthcare.

Our study confirms that there is untapped potential to improve HIV testing rates in China through encouraging CHCs to offer testing. WHO strongly recommends that primary care should provide HIV prevention, therapy and care for members of key populations [26]. Many countries have already included HIV testing as part of screening of key populations within general practice. For example, in the UK, HIV testing is offered by general practitioners in a patient’s initial consultation if they are from an HIV endemic area [27]. In France, offering HIV testing to patients aged 15–70 years with or without high-risk behaviors for HIV is another attempt to increase earlier diagnosis of people with HIV infection [18]. These strategies have proven to be cost-effective [28]. In our study, the attitude of staff in CHCs towards HIV testing appear to be positive with 81% agreeing that HIV testing is an important part of healthcare and those CHCs situated in regions with higher prevalence of HIV (e.g. western) were more likely to have offered HIV testing.

The revamping of CHCs in China and the development of general practice as the “first contact of care in community” has played a key role in the recent healthcare reforms [29]. HIV testing availability is one of the key motivators for having a test done [30]. It seems both acceptable and practical for CHCs to provide HIV testing services based on the wide availability and accessibility of CHCs. In cities, nearly three quarters of CHCs (71%) have been converted from smaller district-level hospitals with on-site capacity for further laboratory investigations (95%) [22]. This may provide a unique opportunity to improve the early detection and management (or at least referrals to appropriate HIV services) for people living with HIV. If an individual is found to be HIV positive and CHC staff are not comfortable in managing HIV, they are encouraged to refer patients beyond their current service capability to hospitals [31]. However, consistent with the financial barriers identified in our study, HIV testing is not currently freely available in primary care and the insurance coverage is fragmented with differing levels of coverage and access to health care by different insurance schemes, leaving gaps that require out-of-pocket payments by patients. This is reflected in our study’s finding that only a quarter of staff in CHCs would offer HIV testing even on request from the patient. The most cited barrier was the staff members’ concerns about the patients’ ability to pay or reimbursement issues related to HIV testing. Further study is required to examine the willingness-to-pay for HIV testing in China and explore the options on how to offer affordable HIV tests within CHCs that may help to increase testing rates especially for members of key populations.

WHO recommends HIV testing and counseling should be offered to people from key populations when they visit a doctor for any health problem [32] or at least once a year for key populations like sexually active men who have sex with men [33]. Our study found that only 6% of CHC staff were involved in providing care to people living with HIV within the preceding month and, a further 10% of them have consulted patients at high risk for HIV in the same period. This suggests that at least some people living with HIV and people belonging to populations at risk for HIV infection are willing to visit primary care doctors in the community. Our findings are consistent with a British study that reported people at-risk for HIV infection were willing to visit their general practitioners as their first point of contact for healthcare [34]. The advantage of incorporating HIV testing into CHCs is that they are closer to where people live and, if the service is integrated into routine care, it could help normalize HIV testing and other services for key populations.

Before encouraging HIV testing through CHCs, several barriers will need to be addressed. We identified concerns from CHC staff over challenges in dealing with the perceived clinical complexities of people from key populations. Nearly half of CHC staff surveyed were also worried that people belonging to key populations might scare other patients away, and about a third were concerned of being infected themselves, despite the risk being extremely low. These fears are consistent with other research findings from China [18]. About half the CHC staff cited lack of training as the biggest barrier against offering HIV testing in CHCs and CME may serve as a useful avenue for further training in HIV testing and counseling. Indeed, we found those who were currently offering HIV testing were those who had already had specific training in HIV. Moreover, part of preparing CHCs to offer HIV testing also lies in ensuring privacy and confidentiality of patients requesting HIV testing. Previous surveys of people from high-risk populations have mentioned privacy concerns around HIV testing [10–12]. In our study, half of the staff thought it was difficult to ensure patient confidentiality within the current environment in their CHC. A protected space that ensures privacy in discussing and testing for HIV within CHCs, as well as improvement in training to ensure professionalism, are required before this service is to be introduced.

The strength of the study was its high response rates from a large number of randomly sampled CHCs in Mainland China. Overall, 84% of chosen CHCs participated, and 89% of staff from the CHCs completed the surveys. This high response rate was attributed to the strong network of general practitioners organized at national and provincial levels, and the follow up from the research team (i.e. telephone calls to the clinicians-in-charge to ensure surveys were completed). Our study findings should be read in light of its limitations. This is a cross-sectional study that provided an overview of the current practices and attitudes of CHC staff in China. There is potential for selection bias which might reduce the generalizability of our findings, but we used a three-stage stratified sampling strategy to minimize this. Further qualitative research is needed to elicit and address specific issues that an individual CHC may have regarding offering HIV testing to their patients, particularly to members of key populations. For instance, we did not distinguish the subpopulations that makes up the category of ‘key populations’ in our survey (i.e. female sex workers, men who have sex with men, intravenous drug users) who have differences in cultural norms and thus the attitude of providers towards each subpopulation may differ accordingly. Nevertheless, our survey uncovered common stigmatizing attitudes expressed by some CHC staff which needs to be addressed if HIV testing is to be encouraged through CHCs. In addition, further study to evaluate the cost-effectiveness of offering HIV testing through CHCs in China would be valuable in providing supporting evidence for encouraging all CHCs to offer a HIV testing service. Our study showed that CHC providers are reporting that members of key populations are attending CHCs, and if CHC staff could target testing to members of these populations, it may be a cost-effective means to provide HIV testing. Further research will be needed to estimate what proportion of CHC attendees belong to key populations. Another potential advantage of integrating HIV testing into CHCs is that it may lessen stigma associated with HIV testing, but it will necessitate broad consensus to develop appropriate protocols and further training for primary care providers. Finally, if a patient tests positive, an appropriate referral to receive ongoing HIV care (i.e. linkage to care) is critical. Our study did not collect information on CHC staff’s willingness to ensure linkage to care, and this is an area that warrants further research.

## Conclusions

This study is the largest national survey conducted in Mainland China to evaluate the capacity for improving HIV testing through CHCs. Although only a minority of CHC staff are currently providing HIV testing, most agree that HIV testing is an important part of healthcare. The main barriers to offering HIV testing in CHCs are related to the reimbursement for testing, lack of training, and some negative attitudes towards members of key populations. Improving HIV training of CHC staff, including addressing stigmatizing attitudes, and improving financial reimbursement may help increase HIV testing coverage in China.

## Supplementary information

**Additional file 1.** Survey instruments.

## Data Availability

The datasets used and/or analysed during the current study are available from the corresponding author on reasonable request.

## Abbreviations

aOR: Adjusted odds ratio
CHC: Community health centre
CME: Continuous medical education
HIV: Human immunodeficiency virus
PLHIV: People living with HIV
VCT: Voluntary counselling and testing
