# Supplementary material for: Opportunities and barriers for providing HIV testing through community health centers in mainland China: a nationwide cross-sectional survey
Source: BMC Infect Dis. 2019 Dec 16;19:1054. doi: 10.1186/s12879-019-4673-0 (PMC6916042; doi:10.1186/s12879-019-4673-0)
Supplement: Supplementary file 1 — Additional file 1. Survey instruments. [file 12879_2019_4673_MOESM1_ESM.docx]

**Additional File 1 Survey instruments**

**FOR KEY INDIVIDUAL OF THE CHC**

***Clinic characteristics and patient demographics – Questionnaire for Key Personnel***

**Administrative district:** _____________

**Registry name: ___________ __________**

**Visiting address: _____________________________________________________**

**Telephone: ___________ __**

**Email of the coordinator: _______ _____**

**Date: _________________**

***Note:***

1. ***Please fill the following form with a true profile of the CHC, which contains no information about the parent hospital and affiliated health service stations.***
2. ***Please do not leave any sections blank. If a section does not apply to you, please indicate that it is not applicable (N/A); if the answer of a question is zero, please fill in “0”. Thank-you for the cooperation!***
3. What is the population size of your catchment area? ______________
4. How many people are you serving in your community health centre (CHC)? _____________, of which the number of migrants is___________.
5. Within area under which your CHC serve, are the following clinical services also available? (Please circle Yes (Y) or No (N) the enter the number on the dotted line)

① Provincial hospitals: Y/N _________

② City hospitals: Y/N _________

③ County hospitals: Y/N _________

④ Township hospitals: Y/N _________

⑤ CHCs: Y/N _________

⑥ Personal clinics: Y/N _________

⑦ Private hospitals: Y/N _____ __

1. Your CHC:

① Converted from Class C hospital

② Converted from Class B hospital

③ Organised under Class B hospital

④ Organised under Class A hospital

⑤ Organised under Business unit

⑥ Organised under by co-op

⑦ Organised by individual

1. Demographics of patients in your CHC:

① What is the mean age of the patients: _____________

② What is the ratio of male to female patients: _____________

1. Does your CHC have the following equipment in each consultation room? (Please circle Yes (Y) or No (N)) Are these equipment in good condition? ( Please circle ‘good’ or ‘bad’ accordingly)

① Stethoscope Y/N Good/Bad Condition

② Ophthalmoscope Y/N Good/Bad Condition

③ Otoscope Y/N Good/Bad Condition

④ Thermometer Y/N Good/Bad Condition

⑤ Blood pressure machine Y/N Good/Bad Condition

⑥ Spotlight for gynaecological examination Y/N Good/Bad Condition

⑦ Computer for administrative purposes Y/N Good/Bad Condition

⑧ Computer for keeping medical records Y/N Good/Bad Condition

1. The following questions are about the service at your CHCs: (Please answer all questions and specify what they are in the space provided)

① Can appointments be made in advance: Yes/No

② Number of opening hour per week: ___________hrs

③Average number of doctors’ consultations per month in the last year: ________

④ Different types of allied health consultations are available: Yes/No

⑤ If the above question was yes, how many allied health consultations (incl. nurse-led clinic) were conducted in the last month: ____ ____

1. How many of the following staff do you have in your CHC working full time? (Please circle Yes (Y) or No (N) and enter the number on the line)

① Full time doctors: Y/N ________

② Visiting/part-time specialists: Y/N ________

③ Dentists: Y/N ________

④ Nurses: Y/N ________

⑤ Dental assistants: Y/N ___ __

⑥ Managers/administrators: Y/N ________

⑦ Front desk clerks/receptionists: Y/N _ ______

⑧ Pharmacists: Y/N ________

⑨ Physiotherapists: Y/N ________

⑩ Psychologist/ Social workers: Y/N ________

⑪ Lab technicians: Y/N ________

⑫ Radiographers: Y/N ________

⑬ Others (please specify what they are and the number): ______________________

1. Are the following facilities available? (Please circle Yes (Y) or No (N) for each)

① Drug dispensing Y/N

② Treatment/wound dressing room Y/N

③ Observation/IV drip room Y/N

④ Inpatient beds Y/N (Please indicate how many: ________)

⑤ Wheelchair access Y/N

⑥ Designated Parking facilities Y/N

⑧ Internet access for staff Y/N

⑨Internet access for patients Y/N

1. The range of clinical services your CHC currently provides (Please circle Yes (Y) or No (N)):

① Chronic disease management Y/N

(e.g. Hypertension, Diabetes, Hyperlipidaemia)

② Common ailments (e.g. cold or diarrhoea) Y/N

③ Traditional Chinese Medicine Y/N

④ Sexual health services Y/N

⑤ Family planning/Reproductive health Y/N

⑥ Maternity and infant healthcare Y/N

⑦ Vaccinations Y/N

⑧ Mental healthcare Y/N

⑨ Others: ________________________________

1. Does your CHC offer testing on-site for the following? (Please circle Yes (Y) or No (N))
2. Blood tests for biochemistry/haematology Y/N
3. X-rays Y/N
4. Doppler or Ultrasound Y/N
5. PAP smear for cervical cancer screening Y/N
6. Microbiology Y/N

e.g. microscopy or culture and sensitivity

1. Rapid pregnancy tests Y/N
2. Chlamydia testing Cell Culture: Y/N PCR: Y/N
3. Gonorrhoea testing Cell Culture: Y/N PCR: Y/N
4. Syphilis: VDRL: Y/N TPHA: Y/N Rapid Test: Y/N
5. Hepatitis testing HepB: Y/N HepC: Y/N
6. Rapid HIV tests Y/N
7. Others (Please Specify): ________________________________
8. Does your CHC have a written policy about procedures for the following? (Please circle Yes (Y) or No (N))
9. Infection Control Y/N
10. Sharps disposal Y/N
11. Not sharing injecting equipment Y/N
12. Waste management Y/N
13. Are the following diagnostic and treatment guidelines available to your CHC either in written or online form? (Please circle Yes (Y) or No (N))

① Chlamydia Diagnosis: Y/N Treatment: Y/N

② Gonorrhoea Diagnosis: Y/N Treatment: Y/N

③ Syphilis Diagnosis: Y/N Treatment: Y/N

④ Hepatitis B Diagnosis: Y/N Treatment: Y/N

⑤ Hepatitis C Diagnosis: Y/N Treatment: Y/N

⑥ HIV Diagnosis: Y/N Treatment: Y/N

1. Does your CHC hold meetings to discuss ways to improve patient care? (Please circle the one that applies)
   1. Never
   2. Yearly
   3. Quarterly
   4. Bimonthly
   5. Monthly
   6. Bi-weekly
   7. Weekly
2. Who is involved in these meetings? (Please circle Yes (Y) or No (N))
3. Managers Y/N
4. Doctors Y/N
5. Nurses Y/N
6. Members of allied healthcare facilities Y/N
7. Receptionists Y/N
8. Are these meetings based on any of the following? (Please circle Yes (Y) or No (N))

① Government directives Y/N

② Data generated from own information system Y/N

③ Local Information (e.g. demand survey/ epidemiological data) Y/N

④ Issues related to incidence of biological risks and individual, family and social vulnerabilities (such as violence, drugs and others) Y/N

⑤ Neighbourhood communities Y/N

1. Will the meetings include any of the following activities? (Please circle Yes (Y) or No (N))

① Organisation of work process and service Y/N

② Case discussion (sentinel events, difficult cases, challenging cases) Y/N

③ Planning/ discussion of therapeutic project Y/N

④ Evaluation of team actions (e.g. audit) Y/N

⑤ Monitoring and analysis of health indicators and information Y/N

⑥ Continuing education Y/N

⑦ Other(s)____________________________________________

***- END OF QUESTIONNAIRE -***

**FOR DOCTORS AND NURSES**

***Clinic characteristics and patient demographics – Questionnaire for Doctors and Nurses***

**Note:**

1. **Please fill the following form with a true profile of the CHC, which contains no information about the parent hospital and affiliated health service stations.**
2. **Please do not leave any sections blank. If a section does not apply to you, please indicate that it is not applicable (N/A); if the answer of a question is zero, please fill in “0”. Thank-you for the cooperation!**

**Your demographics:**

- - - Are you a doctor/nurse? Doctor Nurse
    - What is your age? _______ years
    - Gender: Male Female
    - Ethnicity: Han Minority Other (Please specify: ____________)

1. What qualification did you obtain to practice in this CHC? (Tick one box only)
2. Lower than associate degree
3. Associate degree
4. Graduate degree
5. Graduate degree with postgraduate qualifications
6. What is your registered specialty? ___________________
7. How many years have you practised for?______________ years
8. What board qualification do you have? (for both doctors and nurses)
   1. Consultant
   2. Associate consultant
   3. Resident
9. Do you participate in continuous medical or nursing education? Yes/No
10. Are you involved with planning and implementing meetings related to patient care? Yes/ No
11. What are the issues being discussed at the regular planning meetings? (Please circle Yes (Y) or No (N))
    1. Organisation of work process and service Y/N
    2. Case discussion (sentinel events, difficult cases, challenging cases) Y/N
    3. Planning/ discussion of therapeutic project Y/N
    4. Evaluation of team actions (e.g. audit) Y/N
    5. Monitoring and analysis of health indicators and information Y/N
    6. Continuing education Y/N
    7. Other(s)____________________________________________________
12. How many hours do you spend patient care in a week? ___________ hours

**HIV/STI/Hepatitis care:**

1. In the last month, have you managed patients with the following condition(s)? (Please circle Yes (Y) or No (N))
2. Chlamydia Y/N
3. Gonorrhoea Y/N
4. Syphilis Y/N
5. Hepatitis B Y/N
6. Hepatitis C Y/N
7. HIV Y/N
8. In the last month, have you had the following patients in your CHC? (Please circle Yes (Y) or No (N))
9. Intravenous drug user(s) Y/N
10. Female sex worker(s) Y/N
11. Male sex worker(s) Y/N
12. Men who have sex with men Y/N
13. Transgender people Y/N
14. Have you had training in HIV? (Please circle Yes (Y) or No (N))
15. Never Y/N
16. Pre- and post-test counselling of HIV Y/N
17. Clinical diagnosis of STI Y/N
18. Care of people living with HIV Y/N
19. HIV prevention Y/N
20. Have you had training on common STIs? (Please circle Yes (Y) or No (N))
21. Never Y/N
22. Pre- and post-test counselling of STIs Y/N
23. Clinical diagnosis of STIs Y/N
24. Clinical management of STIs Y/N
25. STI prevention Y/N
26. Partner notification of STIs Y/N
27. What will you do if someone requested a HIV test? (Can tick more than one)
28. Tell them you know nothing about HIV and send them away
29. Tell them to come back to see another colleague at your CHC
30. Tell them to go to hospital
31. Tell them to go CDC
32. Others: ________________________________________
33. What will you do if someone is requesting a STI test? (Can tick more than one)
34. Tell them you know nothing about STIs and send them away
35. Tell them to come back to see another colleague at your CHC
36. Tell them to go to hospital
37. Tell them to go CDC
38. Others: _________________________________________
39. What barriers do you have to providing HIV/STI/Hepatitis testing at your CHC? (Please circle Yes (Y) or No (N))
40. Lack of training Y/N
41. Lack of financial incentive Y/N
42. Lack of support from the senior colleagues or management Y/N
43. Not interested Y/N
44. Others: _________________________________________
45. What are the benefits of providing HIV/STI/Hepatitis testing at the CHC? (Please circle Yes (Y) or No (N))
46. There is nothing to gain Y/N
47. Expand available clinical services at the CHC Y/N
48. Will enhance my personal income Y/N
49. Improve job satisfaction Y/N
50. Earning trust from patients Y/N
51. Others: _________________________________________
52. How do you think support/resources can be provided before HIV/STI/Hepatitis testing can be offered at your CHC? (Please circle Yes (Y) or No (N))
53. More training about HIV/STI/Hepatitis testing Y/N
54. Detailed guidelines and manuals on HIV/STI/ Hepatitis testing Y/N
55. Better support from local hospitals Y/N
56. Direct hotline to specialists Y/N
57. Others: _________________________________________
58. What worries do you have about offering HIV/STI/Hepatitis testing to key populations (i.e. men who have sex with men, people who inject drugs, sex workers and their clients, and transgender people) in your CHC? (Please circle Yes (Y) or No (N))
59. Drive other patients away Y/N
60. The clinic will be too difficult to manage Y/N
61. Get infected by them Y/N
62. Not interested Y/N
63. Hate these people Y/N
64. Others: _______________________________

**Attitudes of testing towards HIV/STIs an key populations: (Please tick one box per line)**

|  | Strongly disagree | Disagree | | Neither agree or disagree | Agree | Strongly agree | Don’t know |
| --- | --- | --- | --- | --- | --- | --- | --- |
| I think routine STI testing is an important part of regular healthcare |  | |  |  |  |  |  |
| I am concerned about cost and reimbursement for STI testing |  | |  |  |  |  |  |
| I am concerned that patients will be offended by being offered routine STI testing |  | |  |  |  |  |  |
| I am comfortable discussing routine STI testing with patients |  | |  |  |  |  |  |
| Language barriers prevent some patients from receiving routine STI testing |  | |  |  |  |  |  |
| Patients often feel like they have to accept routine STI testing |  | |  |  |  |  |  |
| Patients receive adequate pre-test information for routine STI testing |  | |  |  |  |  |  |
| Patients receive adequate post-test information for routine STI testing. |  | |  |  |  |  |  |
| Routine STI testing is voluntary; patients are able to decline screening. |  | |  |  |  |  |  |
| Patients do not expect to be offered routine STI testing |  | |  |  |  |  |  |
| I am concerned that routine STI testing will have a negative effect on patients’ opinions about our clinic. |  | |  |  |  |  |  |
| We have the resources needed to implement STI testing. |  | |  |  |  |  |  |
| It is difficult to provide the privacy needed for routine STI testing. |  | |  |  |  |  |  |
| I think routine HIV testing is an important part of regular healthcare |  | |  |  |  |  |  |
| I am concerned about cost and reimbursement for HIV testing |  | |  |  |  |  |  |
| I am concerned that patients will be offended by being offered routine HIV testing |  | |  |  |  |  |  |
| I am comfortable discussing routine HIV testing with patients |  | |  |  |  |  |  |
| Language barriers prevent some patients from receiving routine HIV testing |  | |  |  |  |  |  |
| Patients often feel like they have to accept routine HIV testing |  | |  |  |  |  |  |
| Patients receive adequate pre-test information for routine HIV testing |  | |  |  |  |  |  |
| Patients receive adequate post-test information for routine HIV testing. |  | |  |  |  |  |  |
| Routine HIV testing is voluntary; patients are able to decline screening. |  | |  |  |  |  |  |
| Patients do not expect to be offered routine HIV testing |  | |  |  |  |  |  |
| I am concerned that routine HIV testing will have a negative effect on patients’ opinions about our clinic. |  | |  |  |  |  |  |
| We have the resources needed to implement HIV testing. |  | |  |  |  |  |  |
| It is difficult to provide the privacy needed for routine HIV testing. |  | |  |  |  |  |  |

|  | Strongly confident | confident | | Neither confident or unconfident | unconfident | Strongly unconfident | Don’t know |
| --- | --- | --- | --- | --- | --- | --- | --- |
| How confident are you to obtain a sexual health history from your patients? |  | |  |  |  |  |  |
| How comfortable are you to ask about sexual orientation from your patients? |  | |  |  |  |  |  |
| How comfortable are you to ask about injecting drug use from your patients? |  | |  |  |  |  |  |
| How comfortable are you to ask about sex work from your patients? |  | |  |  |  |  |  |
| How confident are you to provide pre-test HIV counselling? |  | |  |  |  |  |  |
| How confident are you to provide post-test HIV counselling? |  | |  |  |  |  |  |
| How confident are you in ordering HIV testing? |  | |  |  |  |  |  |
| How confident are you in ordering Hepatitis testing? |  | |  |  |  |  |  |
| How confident are you in ordering STI testing? |  | |  |  |  |  |  |

***- END OF QUESTIONNAIRE -***

**Additional file 1 Table S1 Demographic characteristics of primary care staff in China, by province/municipality**

| **Shanghai** | Total  n/N | % (95% CI) | Doctor  n/N | % (95% CI) | Nurse  n/N | % (95% CI) |
| --- | --- | --- | --- | --- | --- | --- |
| Median age (IQR) |  | 36 (30-43) |  | 40 (34-48) |  | 33 (27-40) |
| Female | 491/569 | 86 (83-89) | 163/241 | 68 (61-73) | 328/328 | 100 (99-100) |
| Highest qualification |  |  |  |  |  |  |
| Graduate degree and higher | 289/576 | 50 (46-54) | 183/248 | 74 (68-79) | 106/328 | 32 (27-38) |
| Title | | | | | | |
| Senior | 22/573 | 4 (2-6) | 21/247 | 9 (5-13) | 1/326 | 0.3 (0-1.7) |
| Intermediate | 273/573 | 48 (43-52) | 174/247 | 70 (64-76) | 99/326 | 30 (25-36) |
| Junior | 241/573 | 42 (38-46) | 46/247 | 19 (14-24) | 195/326 | 60 (54-65) |
| None | 37/573 | 6 (5-9) | 6/247 | 1 (1-5) | 31/326 | 10 (7-13) |
| **Chongqing** | | | | | | |
| Median age (IQR) |  | 32 (26-41) |  | 39 (30-46) |  | 28 (25-35) |
| Female | 201/234 | 86 (81-90) | 69/101 | 68 (58-77) | 132/133 | 99 (96-100) |
| Highest qualification |  |  |  |  |  |  |
| Graduate degree and higher | 65/237 | 27 (22-34) | 37/100 | 37 (28-47) | 28/137 | 20 (14-28) |
| Title |  |  |  |  |  |  |
| Senior | 19/239 | 8 (5-12) | 18/101 | 18 (11-27) | 1/138 | 1 (0-4) |
| Intermediate | 52/239 | 22 (17-28) | 26/101 | 26 (18-35) | 26/138 | 19 (13-26) |
| Junior | 141/239 | 59 (52-65) | 44/101 | 44 (34-54) | 97/138 | 70 (62-78) |
| None | 27/239 | 11 (8-16) | 13/101 | 13 (7-21) | 14/138 | 10 (6-16) |
| **Anhui** |  |  |  |  |  |  |
| Median age (IQR) |  | 33 (29-40) |  | 35 (32-42) |  | 31 (27-37) |
| Female | 234/306 | 76 (71-81) | 79/149 | 53 (45-61) | 155/157 | 99 (95-100) |
| Highest qualification |  |  |  |  |  |  |
| Graduate degree and higher | 124/310 | 40 (35-46) | 90/150 | 60 (52-68) | 34/160 | 21 (15-28) |
| Title |  |  |  |  |  |  |
| Senior | 14/307 | 5 (3-8) | 10/150 | 7 (3-12) | 4/157 | 3 (1-6) |
| Intermediate | 81/307 | 26 (22-32) | 49/150 | 33 (25-41) | 32/157 | 20 (14-28) |
| Junior | 203/307 | 66 (61-71) | 87/150 | 58 (50-66) | 116/157 | 74 (66-81) |
| None | 9/307 | 3 (1-5) | 4/150 | 3 (1-7) | 5/157 | 3 (1-7) |
| **Zhejiang** | Total  n/N | % (95% CI) | Doctor  n/N | % (95% CI) | Nurse  n/N | % (95% CI) |
| Median age (IQR) |  | 35 (30-40) |  | 36 (32-43) |  | 32 (28-37) |
| Female | 495/697 | 71 (67-74) | 217/378 | 57 (52-62) | 278/279 | 100 (98-100) |
| Highest qualification |  |  |  |  |  |  |
| Graduate degree and higher | 408/664 | 61 (58-65) | 258/383 | 67 (62-72) | 150/281 | 53 (47-59) |
| Title |  |  |  |  |  |  |
| Senior | 55/662 | 8 (6-11) | 42/379 | 11 (8-15) | 13/283 | 5 (2-8) |
| Intermediate | 262/662 | 40 (36-43) | 174/379 | 46 (41-51) | 88/283 | 31 (26-37) |
| Junior | 303/662 | 46 (42-50) | 139/379 | 37 (32-42) | 164/283 | 58 (52-64) |
| None | 42/662 | 6 (5-8) | 24/379 | 6 (4-9) | 18/283 | 6 (4-10) |
| **LiaoNing** |  |  |  |  |  |  |
| Median age (IQR) |  | 40 (29-48) |  | 46 (37-53) |  | 32 (26-44) |
| Female | 394/445 | 89 (85-91) | 143/193 | 74 (67-80) | 251/252 | 100 (98-100) |
| Highest qualification |  |  |  |  |  |  |
| Graduate degree and higher | 137/462 | 30 (26-34) | 79/199 | 40 (33-47) | 58/263 | 22 (17-28) |
| Title |  |  |  |  |  |  |
| Senior | 42/461 | 9 (7-12) | 27/200 | 14 (9-19) | 15/261 | 6 (3-9) |
| Intermediate | 159/461 | 34 (30-39) | 81/200 | 41 (34-48) | 78/261 | 30 (24-36) |
| Junior | 243/461 | 53 (48-57) | 82/200 | 41 (34-48) | 161/261 | 62 (55-68) |
| None | 17/461 | 4 (2-6) | 10/200 | 5 (2-9) | 7/261 | 3 (1-5) |
| **Shanxi** |  |  |  |  |  |  |
| Median age (IQR) |  | 36 (29-44) |  | 38 (32-46) |  | 33 (26-40) |
| Female | 371/471 | 79 (75-82) | 152/251 | 61 (54-67) | 219/220 | 100 (97-100) |
| Highest qualification |  |  |  |  |  |  |
| Graduate degree and higher | 223/493 | 45 (41-50) | 143/268 | 53 (47-59) | 80/225 | 36 (29-42) |
| Title |  |  |  |  |  |  |
| Senior | 39/489 | 8 (6-11) | 32/264 | 12 (8-17) | 7/225 | 3 (1-6) |
| Intermediate | 153/489 | 31 (27-36) | 82/264 | 31 (26-37) | 71/225 | 32 (26-38) |
| Junior | 261/489 | 53 (49-58) | 133/264 | 50 (44-57) | 128/225 | 57 (50-63) |
| None | 36/489 | 7 (5-10) | 17/264 | 6 (4-10) | 19/225 | 8 (5-13) |
| **Sichuan** | Total  n/N | % (95% CI) | Doctor  n/N | % (95% CI) | Nurse  n/N | % (95% CI) |
| Median age (IQR) |  | 33 (28-40) |  | 37 (32-44) |  | 30 (26-36) |
| Female | 286/359 | 80 (75-84) | 85/157 | 54 (46-62) | 201/202 | 100 (97-100) |
| Highest qualification |  |  |  |  |  |  |
| Graduate degree and higher | 118/365 | 32 (28-37) | 79/162 | 49 (41-57) | 39/203 | 19 (14-25) |
| Title |  |  |  |  |  |  |
| Senior | 19/359 | 5 (3-8) | 18/160 | 11 (7-17) | 1/199 | 1 (0-3) |
| Intermediate | 107/359 | 30 (25-35) | 63/160 | 39 (32-47) | 44/199 | 22 (17-29) |
| Junior | 212/359 | 59 (54-64) | 66/160 | 41 (34-49) | 146/199 | 73 (67-79) |
| None | 21/359 | 6 (4-9) | 13/160 | 8 (4-13) | 8/199 | 4 (2-8) |
| **Yunnan** |  |  |  |  |  |  |
| Median age (IQR) |  | 32 (26-42) |  | 35 (27-44) |  | 29 (24-39) |
| Female | 346/437 | 79 (75-83) | 117/205 | 57 (50-64) | 229/232 | 99 (96-100) |
| Highest qualification |  |  |  |  |  |  |
| Graduate degree and higher | 105/448 | 23 (20-28) | 71/214 | 33 (27-40) | 34/234 | 15 (10-20) |
| Title |  |  |  |  |  |  |
| Senior | 16/442 | 4 (2-6) | 12/213 | 6 (3-10) | 4/229 | 2 (0-4) |
| Intermediate | 89/442 | 20 (16-24) | 56/213 | 26 (21-33) | 33/229 | 14 (10-20) |
| Junior | 237/442 | 54 (49-58) | 86/213 | 40 (34-47) | 151/229 | 66 (59-72) |
| None | 100/442 | 23 (19-27) | 59/213 | 28 (22-34) | 41/229 | 18 (13-23) |
|  |  |  |  |  |  |  |

**Additional file 2 Table S2**

**2 HIV-related training amongst CHC doctors and nurses, by province/municipality**

| **Shanghai** | Total  n/N | % (95% CI) | Doctor  n/N | % (95% CI) | Nurse  n/N | % (95% CI) |
| --- | --- | --- | --- | --- | --- | --- |
| Pre- and post-test counseling | 157/559 | 28 (24-32) | 78/244 | 32 (26-38) | 79/315 | 25 (20-30) |
| HIV clinical diagnosis | 202/561 | 36 (32-40) | 111/245 | 45 (39-52) | 91/316 | 29 (24-34) |
| Treatments and nursing for PLHIV | 156/560 | 28 (24-32) | 71/244 | 29 (23-35) | 85/316 | 27 (22-32) |
| HIV prevention | 320/574 | 56 (52-60) | 135/245 | 55 (49-61) | 185/329 | 56 (51-62) |
| **Chongqing** |  |  |  |  |  |  |
| Pre- and post-test counseling | 64/223 | 29 (23-35) | 37/95 | 39 (29-49) | 27/128 | 21 (14-29) |
| HIV clinical diagnosis | 80/221 | 36 (30-43) | 45/93 | 48 (38-59) | 35/128 | 27 (20-36) |
| Treatments and nursing for PLHIV | 69/226 | 31 (25-37) | 30/95 | 32 (22-42) | 39/131 | 30 (22-38) |
| HIV prevention | 113/230 | 49 (43-56) | 54/97 | 56 (45-66) | 59/133 | 44 (36-53) |
| **Anhui** |  |  |  |  |  |  |
| Pre- and post-test counseling | 80/297 | 27 (22-32) | 50/143 | 35 (27-43) | 30/154 | 19 (14-27) |
| HIV clinical diagnosis | 95/299 | 32 (27-37) | 66/145 | 46 (37-54) | 29/154 | 19 (13-26) |
| Treatments and nursing for PLHIV | 75/295 | 25 (21-31) | 37/141 | 26 (19-34) | 38/154 | 25 (18-32) |
| HIV prevention | 146/296 | 49 (43-55) | 76/142 | 54 (45-62) | 70/154 | 45 (37-54) |
| **Zhejiang** |  |  |  |  |  |  |
| Pre- and post-test counseling | 232/649 | 36 (32-40) | 164/371 | 44 (39-49) | 68/278 | 24 (20-30) |
| HIV clinical diagnosis | 272/645 | 42 (38-46) | 188/370 | 51 (46-56) | 84/275 | 31 (25-36) |
| Treatments and nursing for PLHIV | 250/644 | 39 (35-43) | 153/368 | 42 (36-47) | 97/276 | 35 (30-41) |
| HIV prevention | 395/654 | 60 (57-64) | 242/376 | 64 (59-69) | 153/278 | 55 (49-61) |
| **LiaoNing** | Total  n/N | % (95% CI) | Doctor  n/N | % (95% CI) | Nurse  n/N | % (95% CI) |
| Pre- and post-test counseling | 154/449 | 34 (30-39) | 61/189 | 32 (26-39) | 93/260 | 36 (30-42) |
| HIV clinical diagnosis | 151/442 | 34 (30-39) | 60/188 | 32 (25-39) | 91/254 | 36 (30-42) |
| Treatments and nursing for PLHIV | 141/445 | 32 (27-36) | 51/187 | 27 (21-34) | 90/258 | 35 (29-41) |
| HIV prevention | 243/450 | 54 (49-59) | 96/191 | 50 (43-58) | 147/259 | 57 (50-63) |
| **Shanxi** |  |  |  |  |  |  |
| Pre- and post-test counseling | 128/468 | 27 (23-32) | 67/258 | 26 (21-32) | 61/210 | 29 (23-36) |
| HIV clinical diagnosis | 137/472 | 29 (25-33) | 74/260 | 28 (23-34) | 63/212 | 30 (24-36) |
| Treatments and nursing for PLHIV | 89/471 | 19 (15-23) | 39/259 | 15 (11-20) | 50/212 | 24 (18-30) |
| HIV prevention | 218/476 | 46 (41-50) | 117/260 | 45 (39-51) | 101/216 | 47 (40-54) |
| **Sichuan** |  |  |  |  |  |  |
| Pre- and post-test counseling | 123/360 | 34 (29-39) | 68/161 | 42 (35-50) | 55/199 | 28 (22-34) |
| HIV clinical diagnosis | 136/358 | 38 (33-43) | 78/160 | 49 (41-57) | 58/198 | 29 (23-36) |
| Treatments and nursing for PLHIV | 133/357 | 37 (32-42) | 63/159 | 40 (32-48) | 70/198 | 35 (29-42) |
| HIV prevention | 199/362 | 55 (50-60) | 99/162 | 61 (53-69) | 100/200 | 50 (43-57) |
| **Yunnan** |  |  |  |  |  |  |
| Pre- and post-test counseling | 247/438 | 56 (52-61) | 115/209 | 55 (48-62) | 132/229 | 58 (51-64) |
| HIV clinical diagnosis | 213/437 | 49 (44-54) | 116/209 | 56 (48-62) | 97/228 | 43 (36-49) |
| Treatments and nursing for PLHIV | 220/434 | 51 (46-55) | 96/207 | 46 (39-53) | 124/227 | 55 (48-61) |
| HIV prevention | 311/441 | 71 (66-75) | 147/212 | 69 (63-75) | 164/229 | 72 (65-77) |
